# Energy and exergy analysis of three leaved yam starch drying in a tray dryer: parametric, modelling and optimization studies

**DOI:** 10.1016/j.heliyon.2022.e10124

**Published:** 2022-08-10

**Authors:** Kenechi Nwosu-Obieogu, Emmanuel Olusola Oke, Simeon Bright

**Affiliations:** a Department of Chemical Engineering, College of Engineering and Engineering Technology, Michael Okpara University of Agriculture, Umudike, Nigeria; b Department of Mechanical Engineering, College of Engineering and Engineering Technology, Michael Okpara University of Agriculture, Umudike, Nigeria

**Keywords:** Energy utilization ratio, Sustainability index, Exergy loss, Exergy efficiency

## Abstract

Engineering conservation during the drying process is paramount as it will help in the preservation and cost minimization of food products during processing to avoid spoilage and maximize their utilization in society. Unlike other yam species, three-leaved yam starch (TLYS) contains phytonutrients for the treatment of ailments such as diabetes and rheumatism. This work examined the energy and exergy of TLYS drying. The starch was extracted from the tuber and dried while the temperature, time, air velocity, and sample thickness were varied. TLYS proximate and SEM analysis revealed a significant amount of starch. Energy analysis revealed that energy utilization (EU) and energy utilization ratio (EUR) increased as the temperature rose and decreased as drying time increased; energy efficiency (EE) increased steadily and then reduced as drying time increased. Exergy analysis revealed that drying temperature increased exergetic efficiency and loss; drying time increased exergetic efficiency from 30 min to 4 h. The highest exergy loss was observed when the sample was dried for 4 h and the thickness is 17 mm; as the thickness decreased to 12.75 mm, the exergy loss decreased from 2.471392 J/s to 1.459247 J/s; the highest exergy efficiency of 2.471392 J/s was observed at the thickness of 4.25 mm, and the sustainability index increased as the sample thickness increased and decreased as the drying air temperature decreased. Response surface methodology (RSM) was utilized to model and optimize the effect of the process’s inherent operating factors (temperature, time, and air velocity) and maximize the process’s energy and exergy efficiency. The (Analysis of Variance) ANOVA revealed a second-order polynomial model with an R^2^ (0.9911), Adj R^2^ (0.9797) and Pred R^2^ (0.8577) for energy efficiency and R^2^ (0.9824), Adj R^2^ (0.9598), and Pred R^2^ (0.7184) for exergy efficiency, indicating a significant correlation between observed and predicted values. At a temperature of 60 °C, a time of 3 h, and an air velocity of 1.5 m/s, the optimal energy efficiency of 75.09 % and exergy efficiency of 99.221% were obtained with desirability of 0.997. The findings of this study can be used to improve the design and development of driers for TLYS preservation.

## 1 Introduction

Bitter yam (*Dioscorea dumetorum*)*,* alternatively known as three-leaved yam, is a member of the *Dioscorea* and *Dioscoreaceae* families (Bai and Ekanayake, 1998). Other names for African bitter yam include wild yellow yam, trifoliate (three-leaved) yam, and cluster yam. In South-eastern Nigeria, it is also known as ‘*Ji una*’ or ‘*Ji ona*’. Bitter yam is high in phytonutrients and is used as a diabetic food and a herbal remedy for a variety of ailments (Oke et al., 2020; Dike et al., 2012; Medoua et al., 2005; Owuamanam et al., 2013). However, despite its potential applications in the bakery and pharmaceutical industries, bitter yam remains an underutilized tropical tuber (Ukpabi, 2010). The bitter taste and high post-harvest hardening of the tubers are two reasons for their underutilization (Medoua et al., 2005).

One of the mediums for three-leaved yam storage and transportation without deterioration is via conversion of dried starch (Guedes et al., 2021). Starch is a complex carbohydrate, (C_6_H_10_O_5_) that is white, powdery and tasteless consisting of 30% amylose and 70% amylopectin. It is found in the cereals, roots and tubers occurring in large quantities such as sweet potatoes (Kolarič et al., 2020), and Chinese yam (Li et al., 2020). Starch is been applied as a raw material in the pharmaceutical, food, cosmetic and chemical industries. It is packaged in powdery or granular form and utilizes drying as a unit operation in its processing (Fan et al., 2021).

Drying is a thermodynamic process comprising heat, mass transfer, and high energy demand for moisture reduction (Yogendrasasidhar and Setty, 2018; Roustapour et al., 2015). The first law of thermodynamics assesses the conservation of energy and analyzes the engineering systems' performance and system loss while the second law of thermodynamics examines energy systems design and improvement and proffers ways of utilizing energy resources efficiently (Dincer, 2002; Akpinar, 2019). Exergy is an effective utilization technique that promotes sustainable development by revealing the possibility of developing energy systems that are efficient by reducing inefficiencies in existing ones (Sahin and Dincer, 2002).

Various researchers have studied the energy and exergy analyses of drying some agricultural products such as green pepper (Akpinar, 2019), cassava starch (Aviara et al., 2014), okra plant (Okunola et al., 2021), tomato slices (Arepally et al., 2017), walnut (Chen et al., 2020) and sweet potato (Kolarič et al., 2020). Their findings revealed a rise in the sustainability index as the exergetic efficiency increased. As a result, modelling and optimization of process parameters aid in characterizing their impact on responses and developing the optimal condition by searching for the most suitable solution to a problem among several alternatives (Benhamza et al., 2021; Zalazar-Garcia et al., 2022).

RSM allows the evaluation of dependent variable data to obtain the equation that models and optimizes the process (Benhamza et al., 2021). According to the literature, only a few studies have used RSM to optimize the energy and exergy efficiency of food drying; Zalazar-Garcia et al. (2022) used RSM to investigate the exergy, energy, and sustainability assessment of integrated convective air-drying with pretreatments to improve the nutritional quality of pumpkin seeds. Benhamza et al. (2021) successfully designed a solar air heater for food drying based on energy, exergy, and improvement potential. Aydoğmuş et al. (2022) studied the isothermal and non-isothermal drying behaviour of grapes via an exergy model using RSM. Mojaver et al. (2019) optimized the exergy analysis of integrated biomass gasification, fuel cell and high-temperature sodium pipe system. The drying behaviour of *Ocimum basilicum Lamiaceae* was successfully analyzed using RSM. Except for Oke et al. (2020), who predicted TLYS drying using soft computing models, there is currently little literature on TLYS drying.

The presence of phytochemicals in TLYS, on the other hand, distinguishes it from other edible starch products and the development of convenient drying conditions to enhance its health-relevant attributes and quality of TLYS for human consumption presents the novelty in this work; thus, this work analyses the moisture content, employs one factor at a time (OFAT) and RSM to determine the optimum operating variables for temperature, time and air velocity in the energy and exergy analysis of TLYS drying. However, the relevance of renewable energy in drying cannot be overemphasized as it encourages sustainability, conserves energy and cost of drying food products; Hence, the need to exploit the objectives of this research for further development of a cost-effective solar drier for TLYS drying (Khanlari et al., 2021; Afshari et al., 2021).

## 2 Materials and methods

### 2.1 Equipment

Electronic compact scale balance (AUW320, Shimadzu, China), Hygrometer (AT-303C.Shangdong, China), Anemometer (AM4206, Delhi, India), Multi Thermometer (ST9283, Mextech, India), Digital Vernier calliper, Blender or grating machine, Muslin cloth, Shaped rectangular plastic plate, Round pan, Electro thermal oven (Heratherm Oven CP 210997, U.S.A), Desiccators (MA-204, Ambala, India).

### 2.2 Experimental procedure

TLY tubers were bought at a local market, washed, and the bark was peeled off. 35 kg of peeled tubers were crushed in a locally made grater. The resulting pulp was mixed with enough water to form a slurry. The fibre was removed from the slurry by sieving it through a 75 mm sieve, and the subsequent starch milk was allowed to settle for 7 h before sieving the supernatant. TLYS (30 g) was moulded with a rectangular thickness of 4.25 mm and weighed with a digital compact scale balance (AUW320, Shimadzu, India). The initial moisture content of the starch was determined in a drying oven at 105° c for 48 h until a constant weight was obtained; the drying experiments were repeated three times; the proximate analysis on the dried sample was performed using (AOAC) Association of Official Analytical Chemist (2003) technique. A micrograph of starch granules was taken using a JSM 35 Genie Scanning Electron Microscope (SEM).

### 2.3 Batch convective drying equipment description and operation

**Figure 1:**
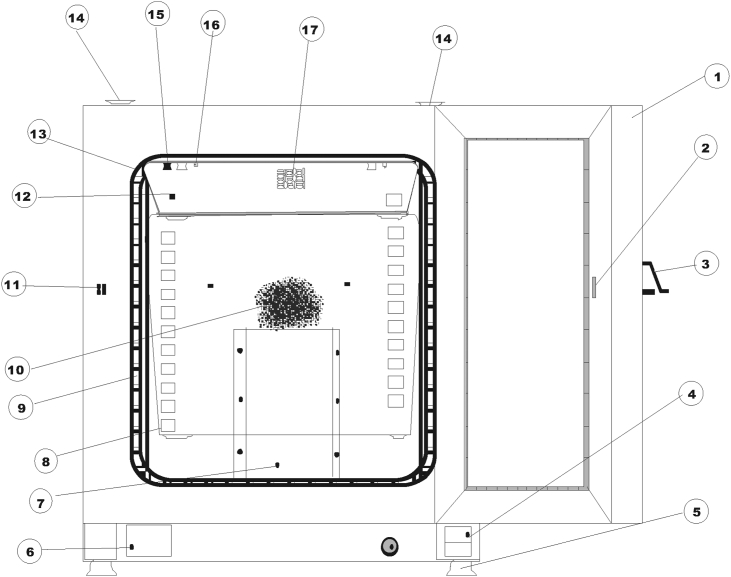
Schematic diagram of the dryer and its features.

The equipment used for the drying was a tray dryer model (Heratherm Oven CP 210997). A diagram of the dryer and its features is presented in Figure 1. It comprises a drying chamber of 360 × 620 × 460 mm with three perforated trays of 327 × 405 mm horizontally placed and vertically stacked. A digital anemometer (Model PM6252A) was used to determine the air velocity inside the chamber and varied it from 1.5 to 3.0 m/s; the relative humidity was between 50 and 60%.

The dryer was preheated to a particular temperature, and 3 TLYS samples moulded in rectangular shape were placed on the trays. A multi-thermometer (Model TA298) was used to evaluate the dry air’s inlet, outlet, ambient temperature, and relative humidity. The features of the dryer depicted in Figure 1 are as follows: (1) Outdoor (2) Door latch cut out (3) Door latch handle (4) Door hinge (5) Levelling foot (6) Nameplate (7) Air battle top piece (9) Shelf support (10) Fan cover, coupled with air baffle (11) Door hook catch (12) Air baffle (13) Door seal (14) Stacking pad (15) Spring for air baffle (16) Temperature detector (17) Air tube for exhaust.

### 2.4 Determination of moisture content

The moisture content of the dried TLYS was evaluated by the gravimetric method (AOAC, 2003) via direct heating in an oven at 105 °C for 48 h presented in Eq. (1).

(1) $$\text{MC ​}=\frac{w_{i}-w_{f}}{w_{f}}\times 100$$

where *MC* is the dry basis (db) moisture content (%); *w*_*i*_ refers to sample weight taken in g for drying at 105 °C, and *w*_*f*_ is the sample weight in g after drying at 105 °C.

### 2.5 Energy analysis

The energy utilization (EU) was determined by using the law of energy conservation presented in Eq. (2) (Aviara et al., 2014):

(2) $$EU={\dot{M}}_{\mathrm{da}}\left(h_{\mathrm{dai}}-h_{\mathrm{dao}}\right)$$

EU = Energy utilized (kJ/s); ${\dot{M}}_{\mathrm{da}}$= dry air mass flow (kg/s), $h_{\mathrm{dai}}$= dry air inlet enthalpy (kJ/kg) and $h_{\mathrm{dao}}$= dry air outlet enthalpy (kJ/kg).

The mass flow rate of air was determined using the formula in Eq. (3).

(3) $${\dot{M}}_{\mathrm{da}}=\rho_{a}V_{a}A_{da}$$

where $\rho_{a}$ is the density of air, *V*_*a*_ is the speed of air inside the dryer, and $A_{da}$ is the air cross-section.

The energy utilization ratio to the given energy in the dryer (Okunola et al., 2021) is determined by Eq. (4).

(4) $$\mathrm{EUR}=\frac{{\dot{M}}_{\mathrm{da}}\left(h_{\mathrm{dai}}-h_{\mathrm{dao}}\right)}{{\dot{M}}_{\mathrm{da}}\left(h_{\mathrm{dai}}-h_{\mathrm{da}\infty }\right)}$$

where $h_{\mathrm{da}\infty }$ is the ambient dry air J/kg enthalpy, EUR is the energy utilization ratio, and ${\dot{M}}_{\mathrm{da}}$ is the mass flow rate of air in kg/s.

Energy efficiency was assessed as the ratio of the energy spent to the energy supplied using Eq. (5)

(5) $$\eta_{en}=\frac{E_{i}-E_{o}}{E_{i}}=\frac{\left(h_{ai}-h_{ao}\right)}{h_{\alpha i}}\times 100$$

▪_en_ is energy efficiency in %, *E*_*i*_ is energy input in J/s, and *E*_*o*_ is energy output in J/s.

Inlet and outlet air enthalpy values equal the sum of enthalpy of dry air and water vapour presented in Eq. (6) (Aviara et al., 2014):

(6) $$h_{\mathrm{da}}=c_{\mathrm{pda}}T+h_{\mathrm{fg}}w$$

where $h_{\mathrm{da}}$ is the dry air enthalpy (inlet/outlet) (kJ/kg); $c_{\mathrm{pda}}$= dry air specific heat (inlet/outlet) (kJ/kg °C); *T* = air temperature (inlet/outlet) (°C); $h_{\mathrm{fg}}$= latent heat of vaporization of water (kJ/kg) and *w* = humidity ratio of air (kg water/kg dry air). Air specific heat is determined from Eq. (7)

(7) $$C_{\mathrm{pda}}=0.0001T+0.9675$$

Humidity ratio was calculated (Okunola et al., 2021) using Eq. (8).

(8) $$w=0.622P-P_{v}$$

where *w* = ratio of humidity; P= pressure of air (kPa) and $P_{v}$= vapor pressure (kPa).

### 2.6 Exergy analysis

The second law of thermodynamics governed exergy analysis determination (Aviara et al., 2014) as shown in Eq. (9):

(9) $$E_{X}=C_{\mathrm{pda}}\left[\left(T-T_{\infty }\right)-T_{\infty }\mathrm{In}\left(\frac{T}{T_{\infty }}\right)\right]$$

Specific heat of air $C_{\mathrm{pda}}$ in Eq. (7) is substituted, and Eq. (10) becomes:

(10) $$E_{X}=0.0001T+0.967\left[\left(T-T_{\infty }\right)-T_{\infty }\mathrm{In}\left(\frac{T}{T_{\infty }}\right)\right]$$

where *E*_*x*_ is air exergy (kJ/s), *T* = inlet/outlet air temperature (°C) and *T*_*∞*_ = ambient temperature (°C), the exergy inflow and outflow at the inlet and outlet temperatures of the drying chamber were calculated using Eq. (8).

Eq. (11) was used to evaluate exergy loss:

(11) $$E_{\mathrm{Xloss}}=E_{\mathrm{Xinflow}}-E_{\mathrm{Xoutflow}}$$

where $E_{\mathrm{Xloss}}$, $E_{\mathrm{Xinflow}}$ and $E_{\mathrm{Xoutflow}}$ are the exergy loss, exergy inflow and outflow.

Exergetic efficiency is the ratio of exergy outflow in the product drying to exergy of the drying air supplied to the system (Castro et al., 2018). The exergy efficiency was determined by the formula below in Eqs. (12) and (13) (Castro et al., 2018):

(12) $$Ex_{\mathrm{eff}}=\frac{E_{\mathrm{Xinflow}}-E_{\mathrm{Xoutflow}}}{E_{\mathrm{Xinflow}}}$$

Or

(13) $$Ex_{\mathrm{eff}}=1-\frac{E_{\mathrm{Xloss}}}{E_{\mathrm{Xinflow}}}$$

The sustainability index of the process was determined by Eq. (14) (Castro et al., 2018):

(14) $$\mathrm{SI}=\frac{1}{1-Ex_{\mathrm{eff}}}$$

### 2.7 Experimental design

Design-Expert version 10 was used to create the experiment, Box-Behnken design implementing response surface technique comprising of three factors and a three-level design was employed. Temperature, time, and air velocity were the three process parameters in this study, with energy and exergy efficiency serving as the response. Table 1 shows the experimental design summary.

**Table 1:** Summary of the experimental factors coding.

Factor	Units	Level		
		−1	0	1
Temperature	°C	60	67.5	75
Time	Hour	3	3.75	4.5
Air velocity	m/s	1.5	1.75	2

## 3 Results and discussion

### 3.1 The proximate analysis

Table 2 shows the results of the TLYS proximate compositions. It reveals that the starch has an ash content (0.25 %), a crude protein content of 5.58 %, fibre, a pH of 5.9, and an amylose content (21.52 %), indicating the presence of starch. As a result, the table shows that TLYS contains sufficient starch for drying. The results are similar to those of a previous study on cassava starch drying by Aviara et al. (2014), where the amylose content is 23.50 %, also reports by Kolarič et al. (2020) and Li et al. (2020) on the starch extracted from sweet potato and Chinese yam indicates that it contains a considerable amount of amylose.

**Table 2:** TLYS proximate analysis.

Parameter	value
Amylose (%)	21.52
pH	5.9
Crude ash (%)	0.25
Crude fat (%)	0.013
Crude protein (%)	5.58
Crude fibre (%)	Nil

**Figure 2:**
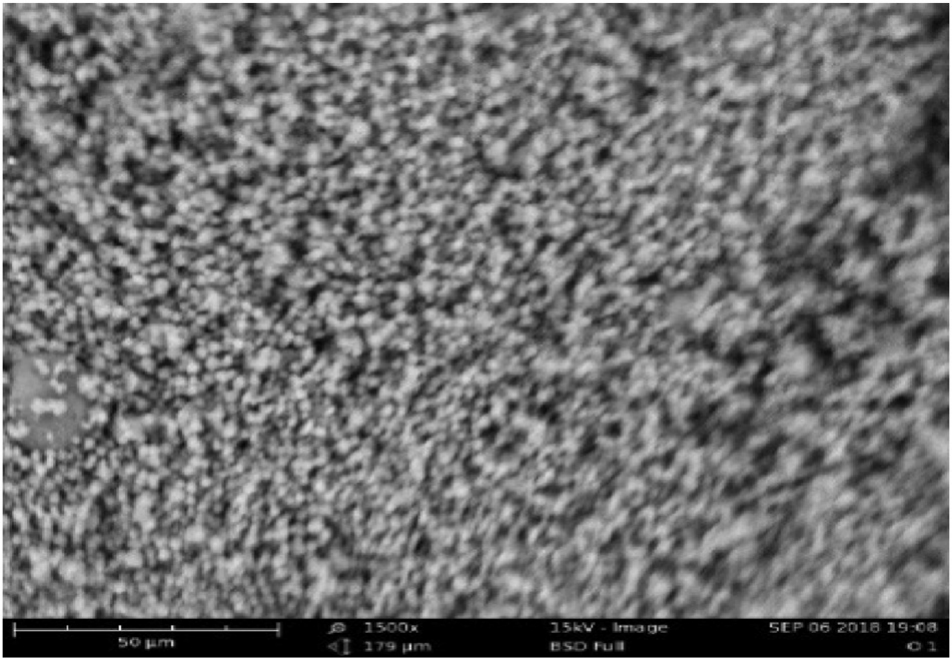
SEM of TLYS.

Figure 2 also depicts the starch’s small granule morphology. The morphology of starch granules is determined by the structure of chloroplasts or amyloplasts (Kaur et al., 2010; Oke et al., 2020). This corroborates with the findings of Li et al. (2020) and Fan et al. (2021) on the morphology of the starch extracted from Chinese yam and sweet potato respectively.

**Figure 3:**
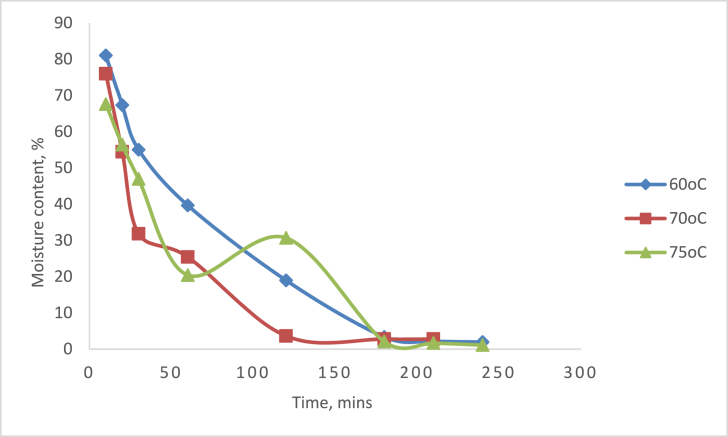
Drying curves of TLYS at different temperatures in a tray dryer.

Figure 3 depicts the variation of TLYS moisture content with time during drying at various temperatures ranging from 60 °C, 70 °C and 75 °C. The moisture content decreased from 81.06% to 1.96 %, 75.98 %–2.79 %, and 67.6 %–1.12 %, at the point where the equilibrium moisture content was obtained for 60 °C, 70 °C, and 75 °C respectively, as the drying time progressed from 10 min to 240 min; similar reports were observed in the drying of cassava (Aviara et al., 2014), Okra plant (Lazarin et al., 2020, Okunola et al., 2021), Guava plant (Guedes et al., 2021), untreated Musa nendra and *Momordica charantia* (Arun et al., 2020), tomatoes (Lopez-Quiroga et al., 2020) and walnut drying (Chen et al., 2020).

### 3.2 Energy analyses

#### 3.2.1 Energy utilization

**Figure 4:**
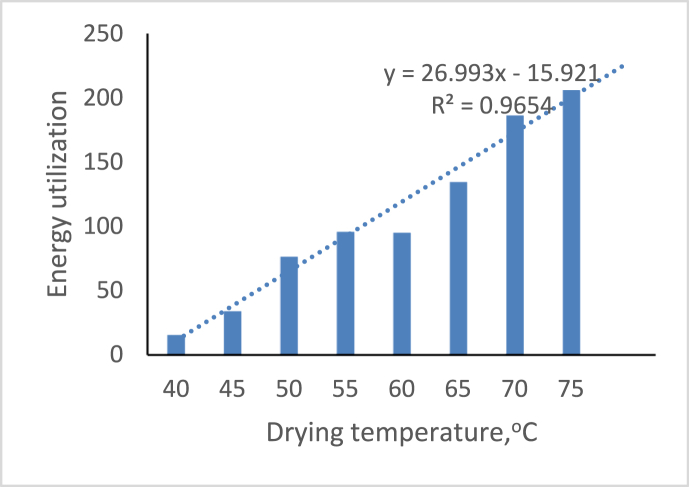
Variation of energy utilization on drying temperature.

The process’s energy utilization (EU) is evaluated using Eq. (2). In addition, the effect of varying drying air temperature and drying time on energy utilization was investigated. The study found that as energy utilization increases, the temperature rises, except for temperatures of 60 °C and 55 °C, which do not differ statistically in energy utilization and may be due to environmental factors affecting moisture content reduction. As a result, the higher the temperature, the more energy consumed. Figure 4 depicts the graph of the effect of drying temperature on energy utilization; energy utilization increased as the drying temperature increased from 40 °C to 75 °C, and the correlation equation yielded a value of R^2^ (0.9654), which is close to 1 and is consistent with Aviara et al. (2014)’s tray dryer study on exergy and energy analysis of native cassava.

**Figure 5:**
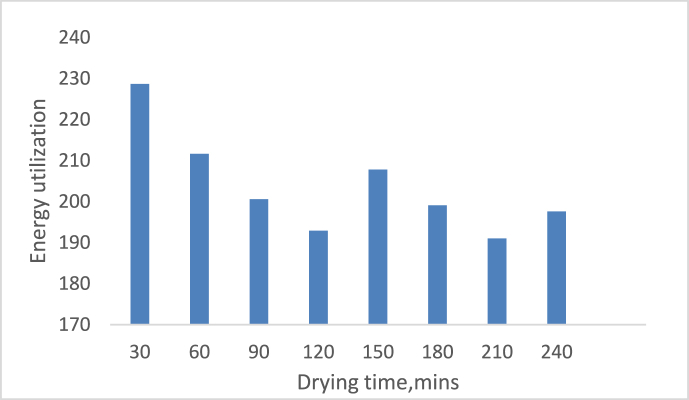
Variation of energy utilization on drying time.

**Figure 6:**
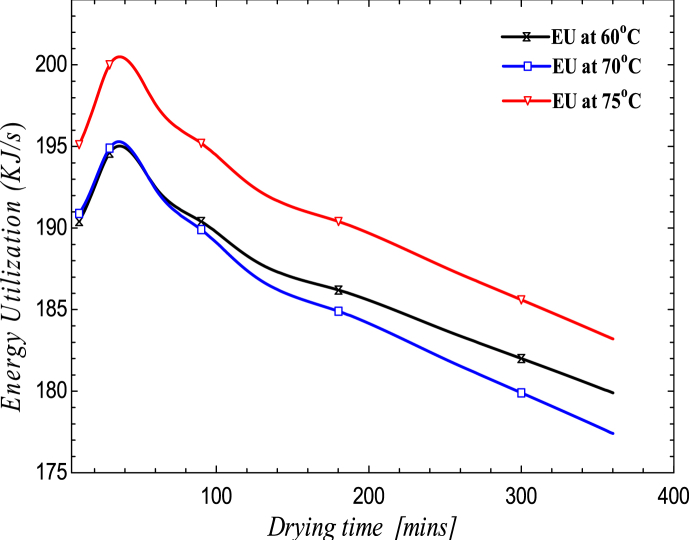
Variation of energy utilization on drying time.

Figure 5 depicts the effect of drying time on system energy utilization. Although the graph was not consistent, it did show a decrease in energy utilization as drying time increased. For example, when the drying time was increased to 150 min, The highest energy was utilized at 30 min, followed by 207.7961 J/s. The EU increases to 195.953, 196.702, and 204.217 J/s for a peak time of 45 min at temperatures of 60 °C, 70 °C, and 75 °C, as shown in Figure 6.

#### 3.2.2 Energy utilization ratio and energy efficiency

**Figure 7:**
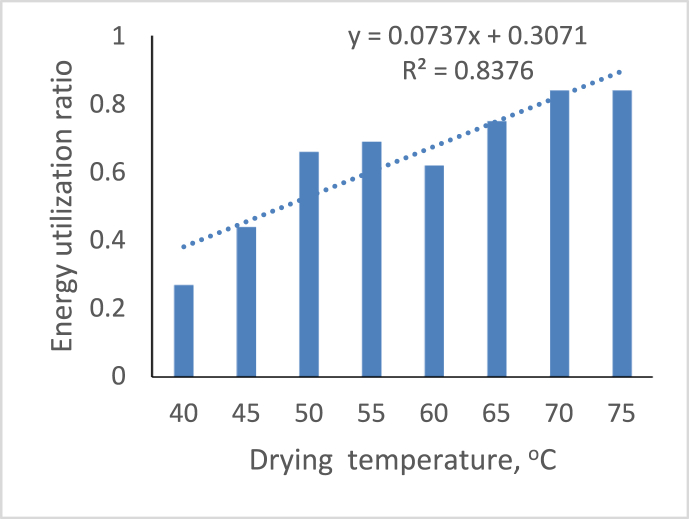
Variation of energy utilization ratio on drying temperature.

Figure 7 depicts a plot for the variation of EUR with temperature; the EUR assessment of three-leaved yam in a tray dryer shows a gradual increase in the EUR from 0.2345 to 0.8012 between 40 °C and 75 °C. At the same time, the air velocity varied between 1.5 and 3 m/s. This is in line with a study (Erbay and Icier, 2009) on drying olive leaves in a tray drier. There exist a linear relationship between temperature(x) and EUR by:

$$y=0.0737x+0.3071\;\text{with}\;\text{an}\;{\text{R}}^{2}=0.8376.$$

**Figure 8:**
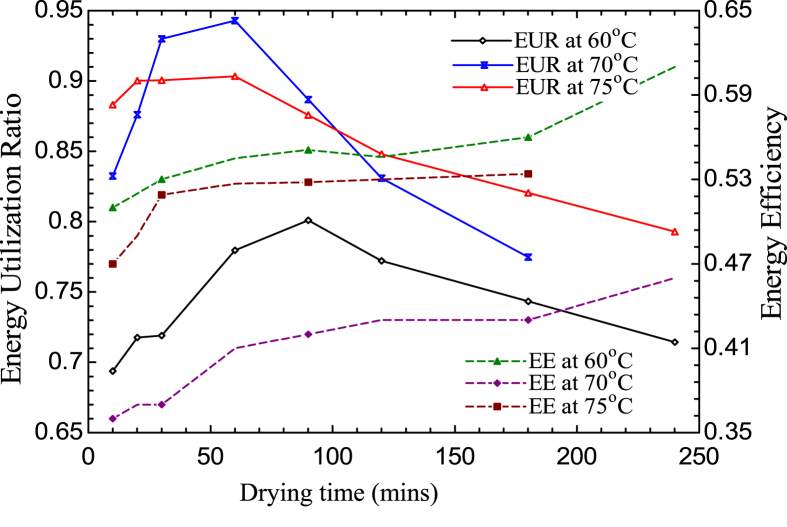
Variation of energy utilization ratio (EUR) and energy efficiency (EE) on drying time.

The effect of the EUR on drying time is quantified in Figure 8; the result shows that the EUR reaches a maximum value of 0.7945, 0.9473, and 0.9001 before gradually decreasing to 0.7945, 0.7723, and 0.8125 after 90, 60, and 58 min of drying time at constant temperatures of 60, 70, and 75 °C, respectively. Minaei (2012) obtained a similar result when drying sour pomegranate arils in a microwave dryer, and (Akpinar, 2005) obtained a similar result when drying potatoes in a cyclone dryer. Figure 8 also shows that the energy efficiency of drying three-leaved yam starch in a tray dryer increased with time at different drying temperatures; the highest values of 0.52(52%), 0.47(47%) and 0.62 (62%) were obtained for drying temperatures of 60 °C, 70 °C and 75 °C respectively. The results for energy utilization, utilization ratio and efficiency is in agreement with the findings of Javed et al. (2020) for hybrid pumped hydro and battery storage for renewable energy-based power supply system, Vilardi et al. (2020) on the energy and exergy analysis of biogas upgrading process and Zhang et al. (2020) on the exergy and energy analysis of pyrolysis of plastic wastes in rotary kiln with heat carrier.

### 3.3 Exergy analyses

#### 3.3.1 Exergy inflow, outflow, loss, efficiency

**Figure 9:**
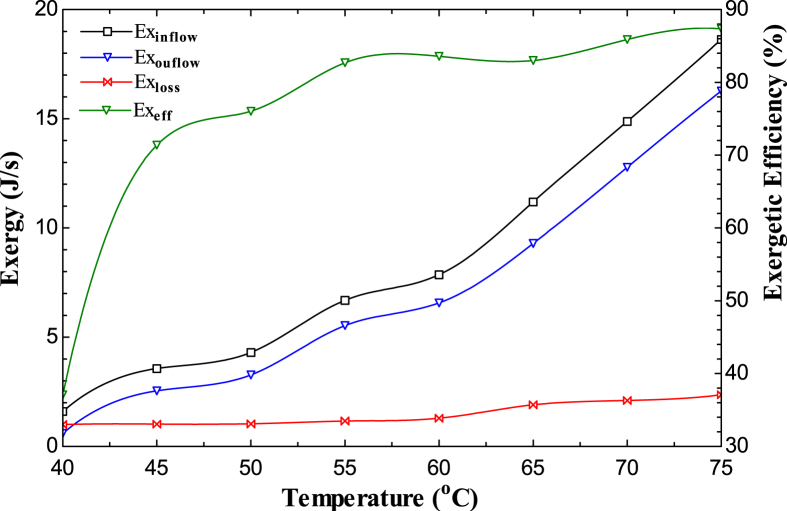
Variation of exergies and Exergetic efficiency on the temperature of the sample.

Figure 9 depicts the variation of the system’s flow exergies; there is an exergy increase for the inflow, outflow, and exergy loss as the temperature rises from 40 °C to 75 °C. Exergy inflow ranges from 1.594 to 18.631 J/s, while exergy outflow ranges from 0.591 to 16.274 J/s. At a temperature of 75 °C, the maximum exergy loss and efficiency were 2.357 J/s and 37%, respectively, at an air velocity of 1.5 m/s. When the system’s exergetic loss improved as the air velocity increased from 2.5 to 3.0 m/s, the exergy loss decreased dramatically. When the sample thickness was 17 mm (the maximum thickness), the exergy loss in the system was observed; as the thickness decreased to 4.25 mm, there was a decline in the exergy loss from 2.471392 to 1.459247 J/s (Vilardi et al., 2020).

Figure 9 depicts the effect of air temperature on the exergy efficiency of the drying system. It demonstrates a moderate increase in the system’s exergy efficiency as the air temperature decreases and vice versa. However, a minor inconsistency occurred between the temperatures 65 °C and 60 °C, increasing from 83.01 to 83.58 % instead of a reduction, but the difference is negligible. These findings are similar to what Akpinar (2004) discovered when drying red peppers in a convection dryer. The system’s exergetic efficiency increases significantly as air velocity increases because increasing air velocity increases the entropy and enthalpy of the input air drier with an increase in exergy efficiency. This finding is similar to what Akpinar (2006) and Nikbakht et al. (2014) discovered when drying strawberries in a silicon dryer and pomegranates in a microwave dryer. The exergy efficiency was 97 % at an air velocity of 2.5 and 3 m/s.

**Figure 10:**
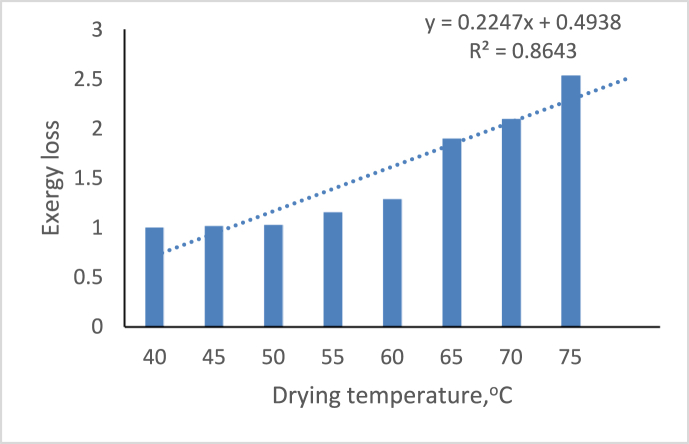
Variation of Exergy loss on drying temperature.

Figure 10 depicts the effect of air temperature on the drying system’s exergy loss. According to the graph, exergy loss decreases as the temperature of the drying system drops, with the highest exergy loss of 2.35676 J/s observed at 75 °C and the lowest exergy loss of 1.003113 J/s observed at 40 °C, implying that the higher the temperature, the greater the exergy loss in the system. This obtained result is similar to the result obtained by Abu-Hamdeh et al. (2020). As a result, the inlet air entering the dryer at a higher temperature has more exergy, increasing moisture evaporation and exergy usage; thus, exergy losses increase (Shaik et al., 2020).

**Figure 11:**
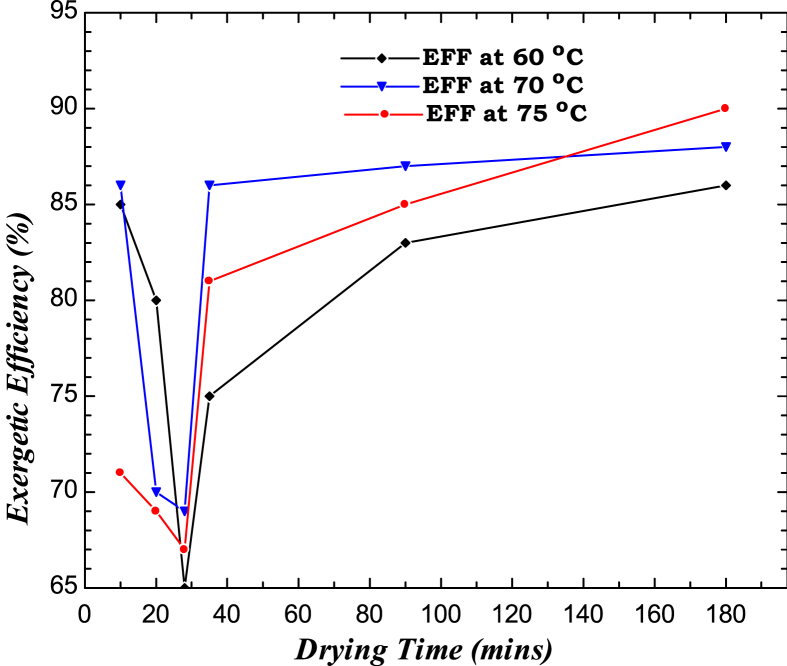
Variation of exergetic efficiency on drying time of sample.

The effect of drying time on exergy losses revealed the highest exergy losses of 1.250799 J/s and 1.333607 J/s at 120 and 150 min, respectively. A decrease of 0.391194 J/s was observed as the drying time exceeded 180 min. At 240 min, it had significantly increased to 0.999293 J/s. Figure 11 depicts the effect of drying time on the drying system’s exergy efficiency. The result, though inconsistent, shows a decrease from 84.05 % to 64 % in the first 27 min and gradually increased up to 92 % at 75 °C. A similar trend was observed at 70 °C but decreased from 86 % and 71 %, respectively, and subsequently increased to 92 % at 4 h drying time. The findings of the exergy analysis (inflow, outflow, loss and efficiency) are consistent with the reports of Shaik et al. (2020) on the exergy and energy analysis of low GWD refrigerants in the perspective of replacement of HFC-134a in a home refrigerator where the drying time and temperature increased with increase in exegetic efficiency and loss.

### 3.4 Sustainability index

**Figure 12:**
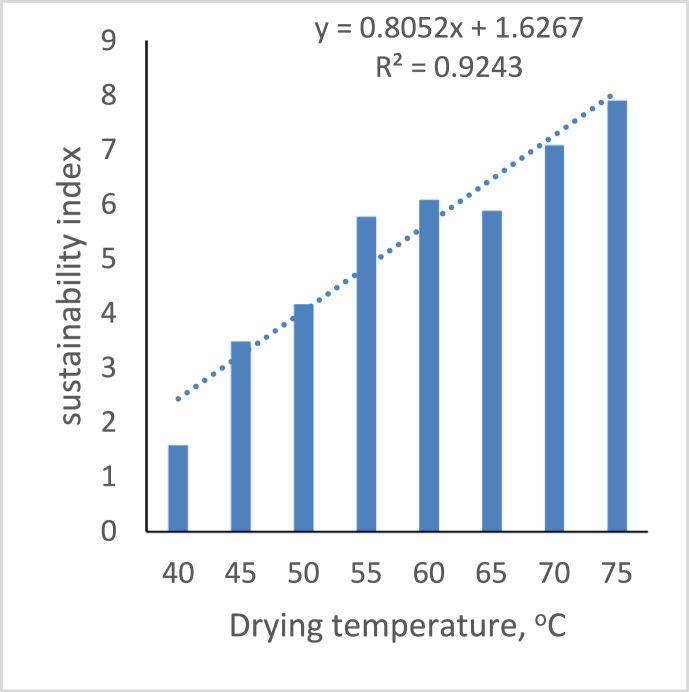
Variation of sustainability index on drying temperature.

**Figure 13:**
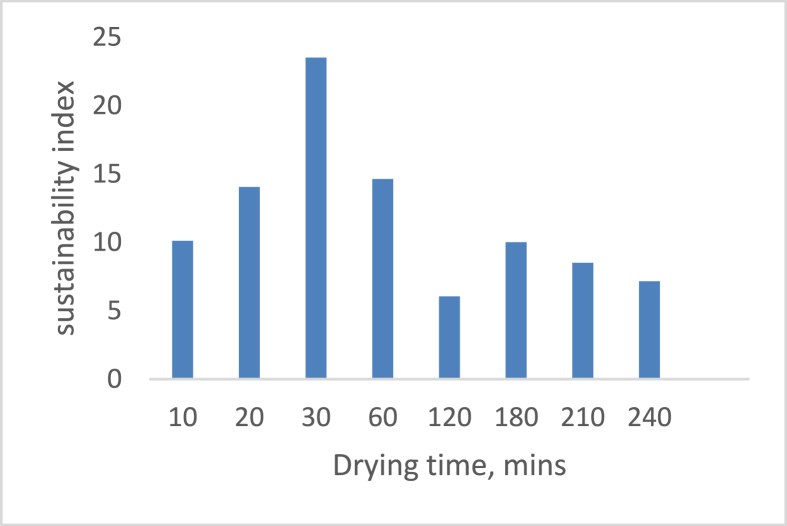
Variation of sustainability index on drying time.

Figure 12 depicts the effect of drying air temperature on the system’s sustainability index. The figure shows that the sustainability index decreases as the drying air temperature decreases; at 65 °C and 60 °C, the values are 5.885 and 6.089, respectively. The variation could be due to environmental conditions on the environment’s ambient temperature and relative humidity. Figure 13 depicts the sustainability index during the drying process. The S.I rises to a maximum of 24.595 in 30 min and gradually falls to 7.518 as the drying time increases up to 4 h these findings coincide with the reports of Javed et al. (2020) on the exergy analysis of hybrid pumped hydro and battery storage for renewable energy-based power supply systems.

**Figure 14:**
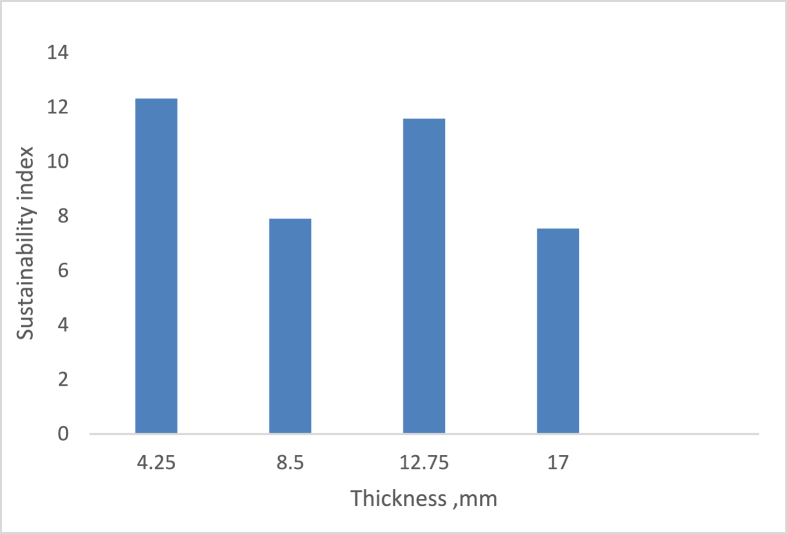
Variation of sustainability index on the thickness of the sample.

Figure 14 depicts the effect of sample thickness on the drying system’s sustainability index. The graph revealed that the highest sustainability index, 12.31198, was obtained at the most negligible sample thickness of 4.25 mm. In contrast, the lowest value, 7.53849, was obtained at the most significant sample thickness of 17 mm (the highest sample thickness). This index factor indicates that the system is sustainable regarding sample thickness variation (James et al., 2020; Hassan et al., 2020).

**Figure 15:**
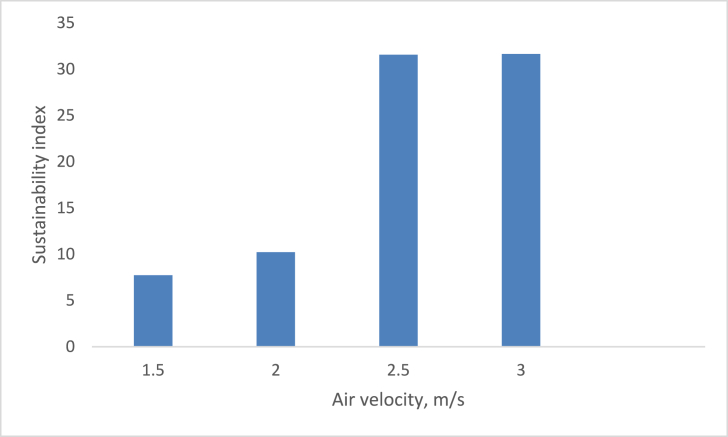
Variation of sustainability index on air velocity.

The effect of air inflow into the drying system on the sustainability index is depicted in Figure 15. The result was found to be inconsistent due to the sinusoidal movement. The system’s air velocity rises, and the sustainability index increases significantly. As the system’s air velocity increased from 1.5 m/s to 3 m/s, the sustainability index increased significantly from 7.717384 to 31.64421. Exergy loss decreased as the temperature of the drying system fell, according to the effect of air temperature on exergy analysis. As the drying system’s air velocity increased, exergy loss and exergetic improvement decreased, while the sustainability index increased (Karthickeyan et al., 2020; Abu-Hamdeh et al., 2020; James et al., 2020).

The experimental design matrix for energy and exergy analysis of TLYS starch drying was analyzed in Design Expert 10.0 using the Box Behnken design consisting of 17 experimental runs. Table 3 shows the experimentally determined response values (energy and exergy efficiency). Maximum energy efficiency was obtained at a temperature of 60 °C, time of 3 h, and air velocity of 1.75 m/s, while exergy efficiency was obtained at a temperature of 67.5 °C time of 4.5 h air velocity of 1.5 m/s. This expanded on the effect of changing process parameters on the energy and exergy efficiency of TLYS starch drying as reported by Benhamza et al., 2021 on the optimization of solar air heaters for food drying based on energy, exergy and improvement potential.

**Table 3:** Experimental run for the energy and exergy efficiency of TLYS drying.

Run	Temperature (°C)	Time (hr)	Air velocity (m/s)	Energy efficiency (%)	Exergy efficiency (%)
1	67.5	3.75	1.75	48.5	84.12
2	60	4.5	1.75	30	81
3	67.5	3	2	53.6	92.9
4	67.5	3.75	1.75	48.5	84.12
5	75	3.75	1.5	42.9	90.8
6	67.5	4.5	2	30.5	88.1
7	60	37.5	1.5	62.3	95
8	60	3	1.75	75.8	94.11
9	67.5	3.75	1.75	48.5	84.12
10	67.5	3.75	1.75	48.5	84.12
11	67.5	3.75	1.75	48.5	84.12
12	67.5	3	1.5	50.13	89.9
13	75	3.75	2	41.3	90.1
14	75	3	1.75	30	81
15	67.5	4.5	1.5	50	96.6
16	60	3.75	2	56.7	93.2
17	75	4.5	1.75	45	90.5

The experimental data’s multiple regression analysis yielded a second order polynomial equation, as shown in Eqs. (15) and (16).

(15) $$energy\;efficiency\;\left(\%\right)=48.50-8.20A-6.75B-2.90C+15.20AB+1AC-5.74BC+0.72A^{2}-4.02B^{2}+1.58C^{2}$$

(16) $$exergy\;efficiency\;\left(\%\right)=84.12-1.11A-0.21B-0.75C+5.65AB+0.77AC-2.87BC+1.72A^{2}-0.82B^{2}+6.94C^{2}$$

where A denotes the temperature (°C), B denotes the time (hours) and C the air velocity (m/s).

In Tables 4 and 5, ANOVA was used to determine the significance of each model and their interactions. Linear terms (A, B, and C), interaction terms (AB and AC), and quadratic terms (B^2^) are significant in the energy efficiency of TLYS starch drying. The model is significant and strong for optimization, with a standard deviation of 1.64, a mean of 47.69, a C.V % of 3.45, an F-value of 86.70, a p-value (0.0001), and an adequate precision (37.126). The model’s lack of fit was 18.91, which is insignificant; this also supports the model’s excellent fit. The coefficient of determination (R^2^) expressed the fitness of the polynomial model, adjusted R^2^ and predicted R^2^, which were obtained as 0.9911, 0.9797, and 0.8577, respectively, indicating the acceptability of the regression model and also, that the experimental data agrees with the predicted data; additionally, for exergy analysis, the overall model is significant, the linear term (A), interaction terms (AB and BC), and quadratic terms (A^2^ and C^2^) have an impact on the exergy efficiency, the model’s significance and adequacy are indicated by the standard deviation of 1.02, mean of 47.69, CV % of 1.15, F-value of 43.31, the probability value of 0.0001, adequate precision of 19.583, lack of fit 7.28, R^2^ of 0.9824, AdjR^2^ of 0.9598, and predicted R^2^ of 0.7184, which is consistent with reports from Zalazar-Garcia et al., (2022) on the improvement of the nutritional quality of pumpkin seeds using RSM and Demirpolat et al. (2022) on the drying behaviour for *Ocimum bassilicum Lamiaceae* with exergy analysis and RSM modelling.

**Table 4:** ANOVA result for the energy efficiency of TLYS starch drying.

Source	Sum of Squares	df	Mean Square	F Value	p-value Prob > F	
Model	2107.77	9	234.20	86.70	0.0001	significant
A-temp	537.92	1	537.92	199.13	0.0001	
B-time	364.91	1	364.91	135.08	0.0001	
C-air velocity	67.45	1	67.45	24.97	0.0016	
AB	924.16	1	924.16	342.11	0.0001	
AC	4.00	1	4.00	1.48	0.2631	
BC	131.91	1	131.91	48.83	0.0002	
A^2^	2.19	1	2.19	0.81	0.3978	
B^2^	68.09	1	68.09	25.20	0.0015	
C^2^	10.49	1	10.49	3.88	0.0894	
Residual	18.91	7	2.70			
Lack of Fit	18.91	7	2.70			Not significant
Pure Error	0.000	4	0.000			
Cor Total	2126.68	10				
Std. Dev.	1.64		R-Squared	0.9911		
Mean	47.69		Adj R-Squared	0.9797		
CV %	3.45		Pred R-Squared	0.8577		
			Adeq Precision	37.126

**Table 5:** ANOVA result for the exergy efficiency of TLYS starch drying.

Source	Sum of Squares	df	Mean Square	F Value	p-value Prob > F	
Model	406.20	9	45.13	43.41	0.0001	significant
A-temp	9.92	1	9.92	9.55	0.0126	
B-time	0.37	1	0.37	0.35	0.5719	
C-air velocity	4.50	1	4.50	4.33	0.0760	
AB	127.80	1	127.80	122.94	0.0001	
AC	2.40	1	2.40	2.31	0.1723	
BC	33.06	1	33.06	31.80	0.0008	
A^2^	12.40	1	12.40	11.93	0.0106	
B^2^	2.81	1	2.81	2.70	0.144	
C^2^	202.72	1	202.72	195.0	0.0001	
Residual	7.28	7	1.04			
Lack of Fit	7.28	3	2.43			Not significant
Pure Error	0.000	4	0.000			
Cor Total	413.47	16				
Std. Dev.	1.02		R-Squared	0.9824		
Mean	88.58		Adj R-Squared	0.9598		
CV %	1.15		Pred R-Squared	0.7184		
			Adeq Precision	19.583		

### 3.5 Optimization of the process conditions

**Figure 16:**
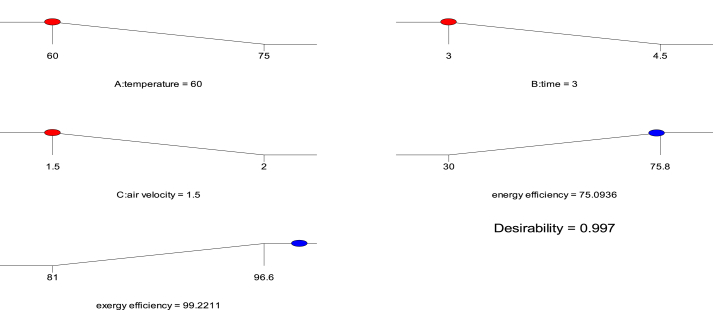
Optimum Conditions for energy and exergy efficiency of TLYS drying.

Figure 16 depicts the optimization of the energy and exergy efficiency of TLYS drying using the Response Surface Methodology (RSM) optimization tool. At a temperature (60 °C), time (3 h), and air velocity (1.5 m/s), the optimal predicted energy efficiency (75.09 %) and exergy efficiency (99.221 %) were obtained with the desirability of 0.997; this agrees with the experimental energy efficiency of 75.8 % and exergy efficiency of 96.6 % obtained with minimum residual errors of 0.71 % and 2.621 %, respectively. Previously, researchers opined that the closer the desirability value is to 1, the better the process’s optimality (Aguele et al., 2021; Mojaver et al., 2019; Aydoğmuş et al., 2022; James et al., 2020).

## 4 Conclusion

The energy and exergy analyses of TLYS drying in a tray dryer at various drying temperatures and times were investigated. As a result, as drying temperature increased, energy utilization, ratio, and energy efficiency increased. Exergy loss and exergetic efficiency, on the other hand, increased with drying temperature. The work’s novelty stems from the fact that TLYS can be used for medicinal purposes as herbs to treat ailments and as a food for diabetics; thus, proximate and SEM analysis has confirmed that it contains a significant amount of starch for this purpose. Furthermore, the ANOVA and RSM optimization results demonstrated that the regression model is acceptable. In addition, the process parameters (temperature, time, and air velocity) had a significant impact on the response (energy and exergy efficiency). For the completeness of this study, further research is required in the design and development of a solar drier for a cost-effective approach to TLYS starch drying.

## Declarations

### Author contribution statement

Kenechi Nwosu-Obieogu: Conceived and designed the experiments; Wrote the paper.

Emmanuel Olusola Oke: Analyzed and interpreted the data.

Simeon Bright: Performed the experiments; Contributed reagents, materials, analysis tools or data.

### Funding statement

This research did not receive any specific grant from funding agencies in the public, commercial, or not-for-profit sectors.

### Data availability statement

Data will be made available on request.

### Declaration of interests statement

The authors declare no conflict of interest.

### Additional information

No additional information is available for this paper.
